# Characterization and Classification of Spanish Honeydew and Blossom Honeys Based on Their Antioxidant Capacity

**DOI:** 10.3390/antiox12020495

**Published:** 2023-02-16

**Authors:** Mónica Fernández-Estellé, Víctor Hernández-González, Javier Saurina, Oscar Núñez, Sonia Sentellas

**Affiliations:** 1 Department of Chemical Engineering and Analytical Chemistry, Universitat de Barcelona, Martí i Franquès 1-11, E08028 Barcelona, Spain; 2 Research Institute in Food Nutrition and Food Safety, Universitat de Barcelona, Av. Prat de la Riba 171, Edifici Recerca (Gaudí), E08921 Santa Coloma de Gramenet, Spain; 3 Serra Húnter Fellow, Departament de Recerca i Universitats, Generalitat de Catalunya, Via Laietana 2, E08003 Barcelona, Spain

**Keywords:** honeydew honey, blossom honey, total phenolic content, total flavonoid content, ferric reducing antioxidant power, PLS-DA

## Abstract

Honey is a very appreciated product for its nutritional characteristics and its benefits for human health, comprising antioxidant, anti-inflammatory, antifungal, and antibacterial activities. These attributes depend on the specific composition of each honey variety, with the botanical origin as one of the distinctive features. Indeed, honeydew and blossom honeys show different physicochemical properties, being the antioxidant capacity, mainly relying on the phenolic compound content, one of the most important. In this work, Folin–Ciocalteu (FC) index, total flavonoid content (TFC), and the antioxidant capacity based on the ferric reducing antioxidant power (FRAP) assay were determined for a total of 73 honeys (50 blossom honeys and 23 honeydew honeys). Mean content of oxidizable species (FC index) ranges from 0.17 to 0.7 mg eq. gallic acid g^−1^, with honeydew honeys being the ones with higher values. Regarding TFC, mean values above 1.5 mg eq. quercetin g^−1^ (method applied in the absence of NaNO_2_) were obtained for honeydew honeys and heather honey. Lower and not discriminatory values (below 0.3 mg eq. epicatechin g^−1^) were obtained in the presence of NaNO_2_. The maximum antioxidant capacity was observed for thyme honeys (2.2 mg eq. Trolox g^−1^) followed by honeydew and heather honeys. Individually, only the FC index was able to discriminate between honeydew and blossom honeys, while the other spectroscopic indexes tested allowed the differentiation of some honey types according to the botanical origin. Thus, a holistic treatment of the results was performed using partial least square discriminant analysis (PLS-DA) for classification purposes using FC, TFC, and FRAP results as data. Honeydew and blossom honey were satisfactorily discriminated (error 5%). In addition, blossom honeys can be perfectly classified according to their botanical origin based on two-class PLS-DA classification models.

## 1. Introduction

Honey is a sweet substance produced by honeybees (*Apis mellifera*), very popular in the human diet, not only because of its taste and nutritional characteristics but also because of their beneficial properties for human health comprising antioxidant, anti-inflammatory, antifungal, and antibacterial activities [1,2,3]. Although carbohydrates and water are the major components in honey, these therapeutic activities are mainly related to other minor components, such as phenolic compounds, amino acids, vitamins, and proteins, among others [4], which occur in different amounts depending on the botanical and geographical origin of honey. The most significant difference is found when comparing blossom or honeydew honeys. Blossom honey is produced by bees from the nectar of flowers, while in honeydew honey, the raw material consists of secretions of living parts of the plants or excretions of plant-sucking insects. Physicochemical properties, as well as the chemical composition, have been observed to be discriminating characteristics between blossom and honeydew honeys [5,6,7,8]. For instance, electrical conductivity (EC) and carbohydrate composition tend to be higher in honeydew honeys than in blossom ones, the carbohydrate profile also being a distinctive feature [8]. Special attention can be paid to the antioxidant capacity of the honeys, which mainly relies on the phenolic compound content. In general, honeydew honeys are characterized by their higher antioxidant activity. In addition, differences within blossom or honeydew subclasses also exist, depending on the botanical and geographical origin [9,10,11,12,13,14]. Because of all the above-mentioned attributes, consumers have established preferences for a specific product. Indeed, the demand for honeydew honey is increasing in many European countries [5]. For that reason, the development of analytical methods to differentiate between honeydew and blossom honeys, but also between varieties within each class, is required to avoid fraudulent practices and to ensure the quality of the products that consumers acquire. 

Several methodologies have been proposed for the discrimination of honeys according to their botanical origin [5]. Apart from the melissopalynological analysis, a tedious and time-consuming technique for the identification of the pollen content, most of the methodologies rely on the determination of different physicochemical parameters and amino acids, sugars, or another family of compound profiling [15]. Among the physicochemical parameters, EC is traditionally used to discriminate between blossom and honeydew honeys. Blossom honeys have EC values of ≤0.8 mS/cm; however, there are exceptions to this general pattern (heather, eucalyptus, lime, and strawberry, among others) [5]. Chromatographic techniques are the techniques of choice for the separation and determination of targeted analytes, with liquid chromatography being the standard approach for compounds such as amino acids, alkaloids, phenolic compounds, or sugars [15,16]. Additionally, gas chromatography (GC) was also appropriate when volatile compounds, organic acids or carbohydrates are used as the markers for differentiating honeys from different origins [15]. Other strategies, which have been extensively used to address food authentication issues, rely on untargeted analysis. For instance, García-Seval et al. developed different untargeted methods based on liquid chromatography with UV detection (LC-UV) or coupled to mass spectrometry (LC-MS) for the characterization and classification of honeys of different botanical origins [17,18,19]. The classification rate using these strategies, especially with LC coupled to high-resolution MS (LC-HRMS), is highly satisfactory, but expensive instrumentation and trained personnel is required. 

As mentioned before, an important honey feature is its high antioxidant activity, mainly due to the presence of phenolic compounds. Identifying and quantifying the main polyphenols, as well as determining the antioxidant capacity, is advisable for a reliable characterization of honeys [9,10,11,12,13,14]. The identification of individual phenolic compounds is an arduous task; for that reason, in general, total phenolic content (TPC), total flavonoid content (TFC), and antioxidant capacity are determined instead using colorimetric assays, which are simple and easy to implement in control laboratories. TPC is usually estimated with the Folin–Ciocalteu (FC) assay [20], although a wide variety of oxidizable compounds interfere with by this determination. Therefore, it is worth to mention that this method is not exclusive for the determination of polyphenolic compounds, other reducing sample components can contribute to the assay response. In contrast, the formation of aluminum complexes is related to flavonoid content [21,22]. Regarding the antioxidant activity, the ferric reducing antioxidant power (FRAP), 1,1-diphenyl-2-picrylhydrazyl (DPPH), and 2,20-azino-bis(3-ethylbenzothiazoline-6-sulfonic acid) (ABTS) assays are among the most extensively applied [23]. 

In the present work, the FC index, the TFC, and the antioxidant capacity were determined for a total of 73 honeys (50 blossom honeys and 23 honeydew honeys), aiming at characterizing honeys according to their botanical origin. Regarding flavonoid content, two different methods, both based on the formation of aluminum chelates, were evaluated, observing that the response of compounds belonging to different flavonoid subfamilies depends on the experimental conditions. Finally, the FRAP method was selected for the determination of the antioxidant capacity. An individual evaluation of the results obtained with the different methods was carried out to ascertain the main characteristics of honeys from different botanical origin. Finally, all the data was treated by means of chemometric tools in order to propose a simple strategy for the classification of honeys according to their botanical origin. 

## 2. Materials and Methods

### 2.1. Reagents and Solutions

Reagents for the determination of spectrophotometric indexes were as follows: FeCl_3_, HCl (37%, *v*/*v*), sodium acetate, and NaOH obtained from Merck (Dramstradt, Germany); Folin–Ciocalteu reagent, NaNO_2_, and Al(NO_3_)_3_·9H_2_O were from Panreac (Barcelona, Spain); formic acid from Sigma-Aldrich (St Louis, MO, USA), Na_2_CO_3_ from Probus S.A. (Badalona, Spain), and Fe (III)-2,2,6-tripyridyl-s-triazine (TPTZ) from Alfa Aesar (Kandel, Germany). Other solvents used were methanol (Fischer Scientific UK Limited, Loughborough, UK), acetonitrile (Panreac, Barcelona, Spain), and purified Milli-Q water (Millipore Corporation, Bedford, MA, USA). 

Gallic acid and epicatechin obtained from Sigma-Aldrich (St Louis, MO, USA), quercetin from Merck (Dramstradt, Germany), and Trolox from Carbosynth (Berkshire, UK) were used for calibration purposes. Stock solution of each standard were prepared in DMSO (Merck, Darmstadt, Germany) at a concentration of 5000 mg L^−1^. 

### 2.2. Instruments and Apparatus

An 8453 UV-Vis Spectrophotometer (Agilent, Santa Clara, CA, USA) was used for the spectrometric determinaton of total phenolic and flavonoid contents, and antioxidant capacity. QS quartz high performance cuvettes (10 mm optical path) from Hellma Analytics (Jena, Germany) were used. Additionally, other laboratory equipment comprises a Vibra Mix R Vortex (Ovan, Barcelona, Spain), a Precisterm thermostatic bath 20L (JP Selecta, Barcelona, Spain) and a bransonic ultrasonic cleaner Branson 5510EMTH (Sigma-Aldrich, MO, USA). 

### 2.3. Samples and Sample Treatment

A total of 73 honeys (50 blossom honeys and 23 honeydew honeys) from several Spanish regions and botanical origins were analysed. All samples were purchased from local markets except two heather honeys that were directly provided by Miel de Braña (León, Spain). Blossom honeys comprised monofloral varieties of orange/lemon blossom (BL), eucalyptus (EU), rosemary (RO), thyme (TH), and heather (HE). Regarding honeydew honeys, holm oak (HO), mountain (MO), and forest (FO) were included in the study (Table 1). 

For the preparation of honey solutions, 1 g of honey was weighted in a 15 mL PTFE centrifuge tube (Serviquimia, Barcelona, Spain) and dissolved in 10 mL of 0.1% formic acid/methanol (80:20, *v*/*v*). The mixture was thoroughly stirred by vortexing and sonicated for 10 min. Honey extracts were kept at 4 °C until use.

### 2.4. Folin–Ciocalteu Index 

The Folin–Ciocalteu (FC) assay was used for an estimation of the oxidizable compounds using Mo(VI) as the oxidant agent, often associated with total phenolic content (TPC). The reaction consisted of mixing 250 µL of honey extracts (or standard solutions) with 250 µL of the FC reagent. After 2 min, 2.5 mL of 7.5% Na_2_CO_3_ were added and the reaction was allowed to develop for 20 min at 40 °C. Afterwards, the reaction was stopped by placing the tubes in an ice bath and the absorbance was measured at 765 nm. Gallic acid standard solutions were prepared in the concentration range from 15 mg L^−1^ to 100 mg L^−1^ in acetonitrile/water (50/50, *v*/*v*) for calibration. Results were expressed as mg of gallic acid equivalents (GAE) as an esimation of the overall concentration of reducing compounds in the honey. 

### 2.5. Total Flavonoid Content (TFC)

The total flavonoid content was determined by aluminum complexation reactions. Two different methods were carried out as follows: 

Method I. Honey extracts (300 µL) or quercetin standard solutions (100 µL) were mixed with 100 µL of 2% Al(NO_3_)_3_ and 100 µL of 1 M sodium acetate. The mixture was then diluted up to 5 mL with acetonitrile/water (50/50, *v*/*v*) and the absorbance was measured at 425 nm. Quercetin standards were prepared in acetonitrile/water (50/50, *v*/*v*) in a concentration range from 100 mg L^−1^ to 1000 mg L^−1^. Results were expressed as mg of quercetin equivalents.

Method II. An aliquot of 200 µL of honey extract (or standard solution) was mixed with 150 µL of 5% NaNO_2_ and 1.5 mL of water. After 6 min, 750 µL of 2% Al(NO_3_)_3_ were added, and the mixture was allowed to react at room temperature for 6 min. Then, 1 mL of 1 M NaOH and water to obtain a final volume of 5 mL were added. The mixture was maintained at room temperature for 15 min, and the absorbance was measured at 510 nm. In this case, epicatechin was used as the standard in a concentration range from 25 mg L^−1^ to 1000 mg L^−1^ prepared in acetonitrile/water (50/50, *v*/*v*). Results were expressed as mg of epicatechin equivalents.

### 2.6. Antioxidant Activity

The determination of the antioxidant activity was performed by the ferric reducing antioxidant power (FRAP) assay following the procedure described by Alcalde B et al. [24]. Briefly, the FRAP reagent was prepared as follows: 20 mmol L^−1^ FeCl_3_, 10 mmol L^−1^ TPTZ (in 50 mmol·L^−1^ HCl) and 50 mmol L^−1^ formic acid solution mixed in the proportion of 1:2:10 (v:v:v). 600 µL of the FRAP reagent were then mixed with 100 µL of the honey extract (or standard) and brought to a final volume of 5 mL with Milli-Q water. After 5 min, the absorbance of the mixture was measured at 595 nm. For calibration, Trolox standards were prepared in acetonitrile/water (50/50, *v*/*v*) in a concentration range from 0.2 mg L^−1^ to 10 mg L^−1^. Results were expressed as mg of Trolox equivalents. 

### 2.7. Data Analysis

Data obtained were subjected to different statistical treatments. ANOVA and t-test statistical analysis to ascertain the significance of the observed differences were carried out with Microsoft Excel (Microsoft, Redmon WA, USA).

In addition, a holistic treatment of the generated data by partial least square discriminant analysis (PLS-DA) was used to address classification issues. The data matrix was constructed using TPC, TFC (methods I and II), and FRAP indexes, expressed as mg of gallic acid, quercetin, epicatechin, or Trolox equivalents, respectively, per g of honey, where each row represents a given sample and each column an antioxidant index. As a result, the matrix dimension was 73 samples × 4 indexes. The SOLO program from Eigenvector Research was used for the chemometric treatment of the data matrix. Prior to PLS-DA, data were preprocessed by autoscaling to equalize the magnitude and amplitude of the variables. 

## 3. Results and Discussion 

### 3.1. Antioxidant Indexes

Aiming at characterizing and classifying different honey samples according to their variety, four different antioxidant indexes were determined for the 73 honey samples (50 blossom honey and 23 honeydew honey) under study. Four spectroscopic methods, namely Folin–Ciocalteu, aluminum complexation (two variants), and FRAP methods, based on different mechanisms, were selected to obtain complementary information. 

**Figure 1:**
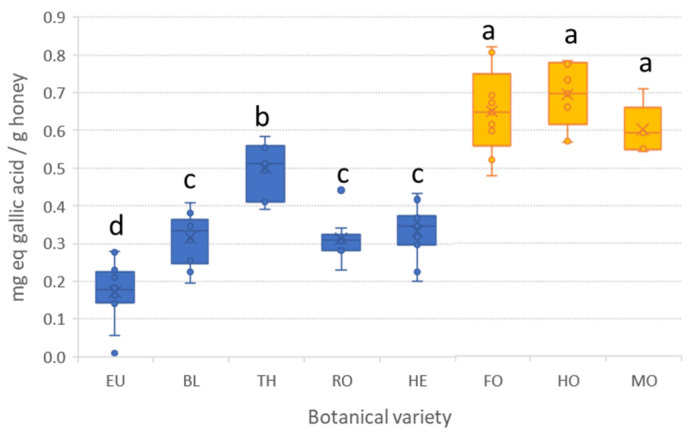
Boxplots with the total content of oxidizable species in honey samples according to the botanical origin. For botanical origin code refer to Table 1. Blue boxes correspond to blossom honeys, while orange ones correspond to honeydew honeys. The same letter indicates no significant differences (α = 0.05).

Firstly, although apart from polyphenolic compounds other species can contribute to the response, the FC method was applied for an estimation of the concentration of total oxidizable compounds, often related with phenolic species. Some variations can be found in the literature regarding the experimental conditions, but the method is always based on the reduction of Mo(VI) to Mo(V) under alkaline conditions. In this work, we accelerated the reaction by conducting the assay at 40 °C. In this way, 20 min were enough for completing the reduction. For quantification purposes gallic acid was used to build the calibration curve, thus, FC index in honey samples was expressed as mg equivalent of gallic acid per gram of honey. The FC data of analyzed honeys is shown in Figure 1, where boxplots with whiskers for each honey variety can be seen. In addition, individual and mean (according to variety) values are given in Table S1 (Supplementary Material). As can be seen in Figure 1, honey samples from a specific botanical origin are quite similar in terms of their phenolic content, showing low data dispersion within groups. Mean FC ranges from 0.17 mg eq gallic acid g^−1^ (EU) to 0.7 mg eq gallic acid g^−1^ (HO), with honeydew honeys being the ones with higher values. Indeed, no significant differences (α = 0.05) were encountered between all the honeydew honeys; however, their FC values were significantly higher than those obtained for blossom honeys. Focusing on the latter, the FC data is highly dependent on the botanical origin. Three groups can be differentiated, with thyme having the highest phenolic content (c.a. 0.5 mg eq gallic acid g^−1^). Honey obtained from orange/lemon, rosemary, and heather nectar showed similar values (around 0.3 mg eq gallic acid g^−1^), and eucalyptus showed the poorest levels. 

**Figure 2:**
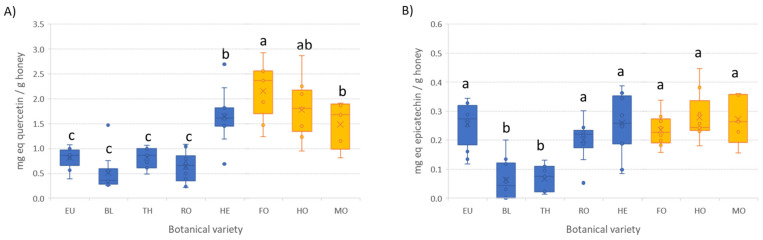
Boxplots with the total flavonoid content in honey samples according to the botanical origin. (**A**) method I (absence of NaNO_2_) and (**B**) method II (presence of NaNO_2_). For botanical origin code refer to Table 1. Blue boxes correspond to blossom honeys, while orange ones correspond to honeydew honeys. The same letter indicates no significant differences (α = 0.05).

The second spectroscopic method applied, relying on aluminum complexation reactions, is reported to determine the total flavonoid content by the formation of Al(III)-flavonoids chelates. However, two different variants were applied, differing in conducting the reaction in the presence or the absence of NaNO_2_, and the wavelength of the maximum absorbance of the formed complex. Although each procedure seems to be adequate for determining a specific subfamily of compounds and, thus, both are inefficient for the evaluation of total flavonoids [22], scientists usually select one of them to have an idea of the flavonoid content in food matrices. In the present study, we applied both methods, namely, here, method I (without NaNO_2_) and method II (with NaNO_2_), to obtain a more complete overview. Prior to the analysis of honey samples, both methods were tested with selected flavonoid standards to evaluate the adequacy to form Al(III)-flavonoid complexes depending on the flavonoid subfamily (see Figure S1 in the Supplementary Material). As can be seen, method I (absence of NaNO_2_) is suitable for flavonols and flavones, while method II (presence of NaNO_2_) works better for catechins (flavanols subfamily). However, the response obtained for the flavanones tested (hesperidin, naringenin, and naringin) is very poor in both methods. In this study, for method I, quercetin was used as standard, and the results were expressed as mg equivalent of quercetin per gram of honey. In the case of method II, epicatechin was selected for quantification purposes and the results were expressed as mg equivalent of epicatechin per gram of honey. The outcomes are depicted in the boxplots with whiskers in Figure 2 and in Table S1 (Supplementary Material). The comparison of both methods (with and without NaNO_2_) reinforces the above-mentioned conclusion that non-equivalent results were obtained, indicating that different features are measured depending on the conditions applied. Honeydew honeys, together with heather honey (blossom honey) resulted in mean values above 1.5 mg eq. quercetin g^−1^ (absence of NaNO_2_), while the rest of blossom honeys contained significantly lower amounts (between 0.50 and 0.82 mg equivalent quercetin g^−1^). Despite TPC, in this case, the flavone plus flavonol content in heather variety honeys is closer to honeydew honeys than to the other blossom ones. This finding was also observed when liquid chromatography with UV detection or coupled to mass spectrometry was used for sample analysis focused on classification purposes [17,18,19]. Although heather honey can be classified into the blossom honey group, its properties and physicochemical characteristics are close to those of honeydew honeys [5,6,8,25]. On the contrary, the flavanol content determined when the Al complexation reaction was performed in the presence of NaNO_2_ (method II) was, comparatively, much lower (below 0.3 mg eq. epicatechin g^−1^). In addition, only orange/lemon and thyme honey varieties displayed levels significantly lower than the other varieties. These findings point out that flavanol content in honey is poor and that cannot be considered a discriminant parameter among varieties.

**Figure 3:**
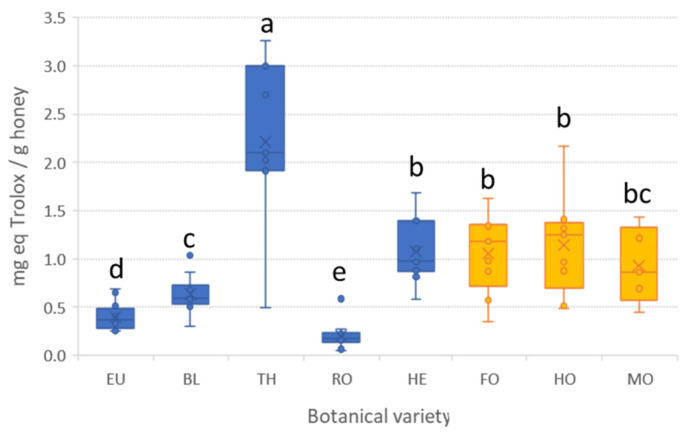
Boxplots with the antioxidant activity (FRAP assay) in honey samples according to the botanical origin. For origin code refer to Table 1. Blue boxes correspond to blossom honeys, while orange ones correspond to honeydew honeys. The same letter indicates no significant differences (α = 0.05).

Finally, the antioxidant activity was determined using the ferric reducing antioxidant power (FRAP) assay based on the reduction of Fe(III) to Fe(II). Mean FRAP values ranged from 0.2 mg eq. Trolox g^−1^ (RO) to 2.2 mg eq. Trolox g^−1^ (TH) (Figure 3 and Table S1 in the Supplementary Material). As can be seen, the antioxidant activity by the FRAP assay depended on the specific botanical origin. Honeydew honeys together with heather honey (blossom honey) exhibited similar antioxidant activity (around 1 mg equivalent Trolox g^−1^). Again, as mentioned before, honeys of heather origin shared characteristic properties with those of honeydew origin. Regarding blossom honeys, they do not follow a specific pattern. Indeed, each type of honey displays distinctive values, with thyme honeys being the ones with maximum antioxidant activity (even higher than those observed for honeydew honeys) followed by orange/lemon, eucalyptus, and, finally, rosemary. These results suggest that the antioxidant activity, measured by means of the FRAP assay, can be useful for discriminating some honeys according to the botanical origin. Indeed, centering the attention to blossom honeys, significant differences (α = 0.05) were observed between varieties. 

### 3.2. Classification of Honeys by PLS-DA

**Figure 4:**
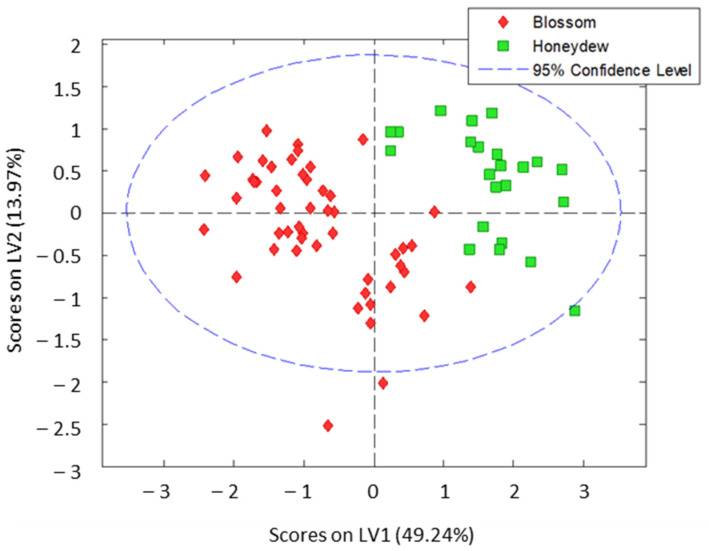
PLS-DA results for the classification of honeys according to both blossom or honeydew origin. Three latent variables were used to build the model.

The individual evaluation of the results obtained with the different indexes allowed the differentiation of some honey varieties. FC data clearly differentiate between honeydew and blossom honeys, but the differentiation of the botanical origin within each type of honey is not possible. On the contrary, the antioxidant activity (FRAP) allowed the differentiation of blossom honeys according to their botanical origin, while heather (blossom) honeys have similar response as honeydew honeys. Regarding the overall concentration of oxidizable compounds, the results obtained showed discrimination only between some varieties. Thus, none of the tested indexes is enough for a total discrimination, honeydew versus blossom honeys and botanical origin within subclasses. Thus, aiming at evaluating the possibility of using these spectroscopic indexes as descriptors for the differentiation of blossom and honeydew honey and, more specifically, to discriminate between different botanical origins, a holistic treatment of the results was performed using chemometric tools. A preliminary model using PLS-DA was built considering the eight individual botanical origins studied. As expected, samples were not totally separated according to their botanical origin. However, both groups of blossom and honeydew honeys were clustered separately in the score plot of LV1 versus LV2 (Figure S2, Supplementary Material), except for heather honeys which were located closer to honeydew honeys. Interestingly, sample classes were here much more separated than in previous studies using LC-UV or LC-MS fingerprints for classification purposes [17,18,19]. In addition, thyme blossom honeys, which exhibited the maximum antioxidant activity (FRAP assay), were discriminated also from the rest of blossom honeys. Indeed, the PLS-DA model constructed considering only two different classes (blossom and honeydew types) showed very promising results (Figure 4), with almost a total discrimination between classes (classification errors of 1 % and 2% for calibration and cross-validation, respectively). 

In view of these results, a classification based on a two-class PLS-DA model was assessed in a more realistic situation. For such a purpose, the set of samples was divided randomly into two subsets. The first one, comprising 60% of the samples, was used for calibration, while the rest 40% of the samples were included in the prediction subset. 

**Figure 5:**
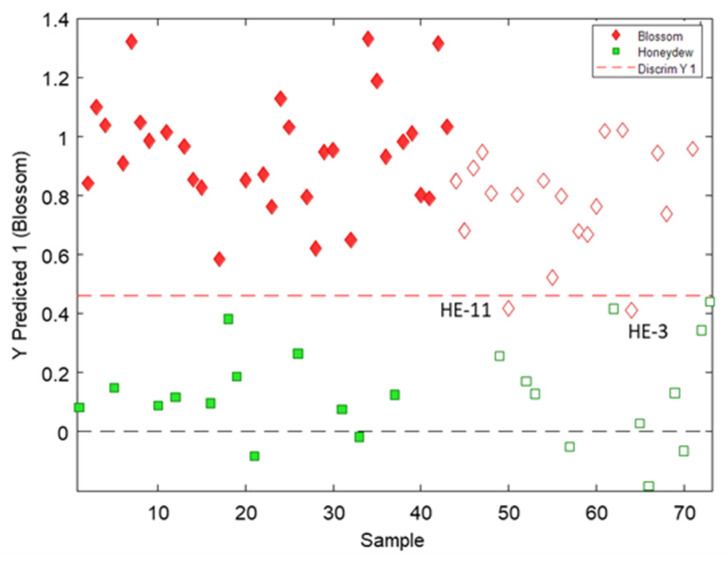
Two-class classification models obtained by PLS-DA of blossom versus honeydew honeys. The calibration set (filled symbols) comprises the 60% of all samples randomly chosen and the prediction set (empty symbols) comprises the 40% remaining samples.

As can be seen in Figure 5, good discrimination between blossom and honeydew honeys was obtained with a prediction error of 5.2% (sensitivity and specificity results for calibration and validation are given in Table S2 of the Supplementary Material). It is worth mentioning that the only two samples that are wrongly classified correspond to heather honeys which displayed, as mentioned above, features closer to those of honeydew honeys than to blossom counterparts. In addition, the separation of heather variety honeys from honeydew honeys was hardly accomplished with more sophisticated methods based on LC with UV detection or coupled to mass spectrometry, where they were mainly classified into honeydew honeys when PLS-DA models were constructed considering all the studied varieties [17,18,19]. Thus, excellent results were obtained here, demonstrating the potential of the proposed strategy for differentiating blossom and honeydew honeys. 

In addition, as an attempt to go further, the possibility of differentiating honeys from different botanical varieties within each type of honey (blossom or honeydew) considering their antioxidant activity was evaluated. Regarding honeydew honeys, the classification errors obtained with the PLS-DA model constructed considering only forest, holm oak and mountain varieties are between 30% and 60%, which highlight the overly similar properties of honeydew honeys obtained from different botanical origins (Figure S3a, Supplementary Material). As a result, although honeydew honeys can be differentiated from blossom honeys using simple descriptors such as antioxidant indexes, these descriptors did not allow the differentiation between the different botanical honeydew honey origins. The exact composition of these honeys is unknown and probably, as the results found point out, some of the plant species used by bees to produce the honeydew honeys evaluated in this study are the same, irrespective of the type of honey. Indeed, this differentiation was either accomplished by non-targeted LC-UV [18], and only data obtained by LC-MS analyses allowed a reliable classification of honeydew honeys [17,19]. However, LC-MS is often not available in control laboratories and requires experienced personnel. 

Finally, the classification of blossom honeys according to each botanical origin was also evaluated separately using multi-class PLS-DA modeling. In this case, promising results were observed in the scatter plot of LV1 versus LV2 (Figure S3b, Supplementary Material). Thyme and heather honeys were separated from the other classes, and the rest of the varieties (BL, EU, and RO), although grouped in the same area, were not strongly overlapped. In addition, samples HE-2 and TH-5, were far away from their corresponding groups, probably because of a wrong assignation or analysis. Thus, these two samples were considered outliers for further studies. 

A classification tree was designed, based on successive two-class PLS-DA models and the classification rate was evaluated. Unfortunately, the number of available samples was not enough to be divided into two subsets (to perform calibration and external prediction), so that a cross-validation was used instead. The first model was constructed comparing thyme origin samples, which belong to the most different class, and the rest of the samples (grouped as others). As can be seen in Figure S4 (Supplementary Material), all the samples are correctly classified. Once the assignations were made, the following model was constructed using only those samples classified as “others” (irrespective of whether the classification is correct or not). In the new PLS-DA model, heather samples were compared to the rest of the samples (grouped again as others). The third method was constructed considering orange/lemon versus eucalyptus and rosemary (as a group). The classification process finished when the model eucalyptus versus rosemary was built. The results for the classification tree are highly satisfactory, with a correct assignation in most of the cases (Table 2). Honeys from thyme, heather, and orange/lemon were perfectly assigned to their corresponding group. However, two eucalyptus and one rosemary honeys were misclassified. 

## 4. Conclusions

Colorimetric assays for the determination the Folin–Ciocalteu (FC) index, the total flavonoid content (TFC), and the antioxidant capacity are fast methods that can be easily applied in control laboratories. The demand for reliable methods for the characterization and classification of honeys from different origins is nowadays increasing, especially for the differentiation of honeydew honey, which is very appreciated among consumers due to its characteristic properties. Considering only the FC results, honeydew and blossom honeys can be easily differentiated; however, the classification of honeys from a specific botanical origin was not feasible. Similarly, the flavone plus flavonol content, determined by the aluminum complexation method in the absence of NaNO_2_, separates honeydew honeys from blossom honeys, except for the heather variety, which, although its origin is floral, values closer to honeydew varieties were obtained. Again, differentiation within honeydew or blossom subsets was not possible. Regarding the aluminum chelation in the presence of NaNO_2_ which assess flavanol content, the results were not conclusive at all, with no significant differences between classes. The antioxidant capacity measured by the FRAP method, discriminates floral varieties, with heather again being closer to honeydew honeys. Analyzing these results, the FC assay seems to be the parameter more effective for discrimination between honeydew and blossom honeys, while the FRAP method allowed the differentiation of blossom honeys according to their botanical origin, but, in this case, heather honey (blossom honey) cannot be discriminated from honeydew honeys. More satisfactory classifications can be obtained with the holistic treatment, by means of PLS-DA models, of the information extracted from the four methods. Firstly, the discrimination between honeydew and blossom honeys were accomplished with errors of around 5% (two heather variety samples were classified as honeydew honeys). Regarding the differentiation of honey within each type of honey (blossom or honeydew), different outcomes were obtained. While honeydew varieties cannot be differentiated by PLS-DA using their FC, TFC, and antioxidant capacity, blossom honeys can be perfectly classified based on two-class PLS-DA classification models. Summarizing, in this manuscript, a simple method (based on spectroscopic determinations), has been demonstrated to be reliable for the differentiation of honeys according to their botanical origin, obtaining similar, or even better results, to the ones reported using more sophisticated techniques such as LC-UV or LC-MS. 

## Figures and Tables

**Table 1:** Blossom and honeydew honeys analyzed.

Honey Type	Variety	Code	No. of Samples
Blossom honey	Eucalyptus	EU	12
	Orange/lemon	BL	10
	Thyme	TH	7
	Rosemary	RO	10
	Heather	HE	11
Honeydew honey	Forest	FO	9
	Holm oak	HO	9
	Mountain	MO	5

**Table 2:** Results from two-class classification models obtained by PLS-DA according to the calibration tree.

Model	Number of LV	Calibration		Validation	
		Sensitivity	Specificity	Sensitivity	Specificity
TH vs others	1	1	1	1	1
HE vs others	1	1	1	1	1
BL vs others	2	1	0.96	1	0.91
EU vs RO	2	1	1	0.91	1

## Data Availability

Data is available upon request to the authors.

## Supplementary Materials

The following supporting information can be downloaded at: https://www.mdpi.com/article/10.3390/antiox12020495/s1, Figure S1: Comparison of the two aluminum complexation methods (with and without NaNO_2_) using standards (500 mg L^−1^) of compounds of different flavonoid subfamilies. Results are expressed as the absorbance measures at the corresponding wavelengths; Figure S2: PLS-DA result for the multi-class classification of honeys according to their variety of origin. Three latent variables were used to build the model; Figure S3: PLS-DA result for the multi-class classification of honeys according to their variety of origin. (A) honeydew honey and (B) blossom honeys. In both cases two latent variables were used to build the model; Figure S4: Two-class classification models obtained by PLS-DA according to the classification tree stablished. (A) thyme vs. others; (B) heather vs. others; (C) orange/lemon vs others; (D) eucalyptus vs. rosemary; Table S1: Antioxidant indexes determined for the 73 samples under study; Table S2: Results from two-class classification models obtained by PLS-DA. The calibration set comprises the 60% of all samples randomly chosen and the prediction set the 40% remaining samples.
