# Supplementary material for: Characterization and Classification of Spanish Honeydew and Blossom Honeys Based on Their Antioxidant Capacity
Source: Antioxidants (Basel). 2023 Feb 16;12(2):495. doi: 10.3390/antiox12020495 (PMC9952669; doi:10.3390/antiox12020495)
Supplement: Supplementary file 1 [file antioxidants-12-00495-s001.zip › antioxidants-2206530-SI.pdf]

antioxidants

Supplementary Material

Characterization and Classification of Spanish Honeydew and Blossom Honeys Based on Their Antioxidant Capacity

Mónica Fernández-Estellé [1], Víctor Hernández-González [1], Javier Saurina [1,2], Oscar Núñez [1,2,3] and Sonia Sentellas [1,2,3,*]

[1] Department of Chemical Engineering and Analytical Chemistry, Universitat de Barcelona, Martí i Franquès 1-11, E08028 Barcelona, Spain
[2] Research Institute in Food Nutrition and Food Safety, Universitat de Barcelona, Av. Prat de la Riba 171, Edifici Recerca (Gaudí), E08921 Santa Coloma de Gramenet, Spain
[3] Serra Húnter Fellow, Departament de Recerca i Universitats, Generalitat de Catalunya, Via Laietana 2, E08003 Barcelona, Spain
* Correspondence: sonia.sentellas@ub.edu

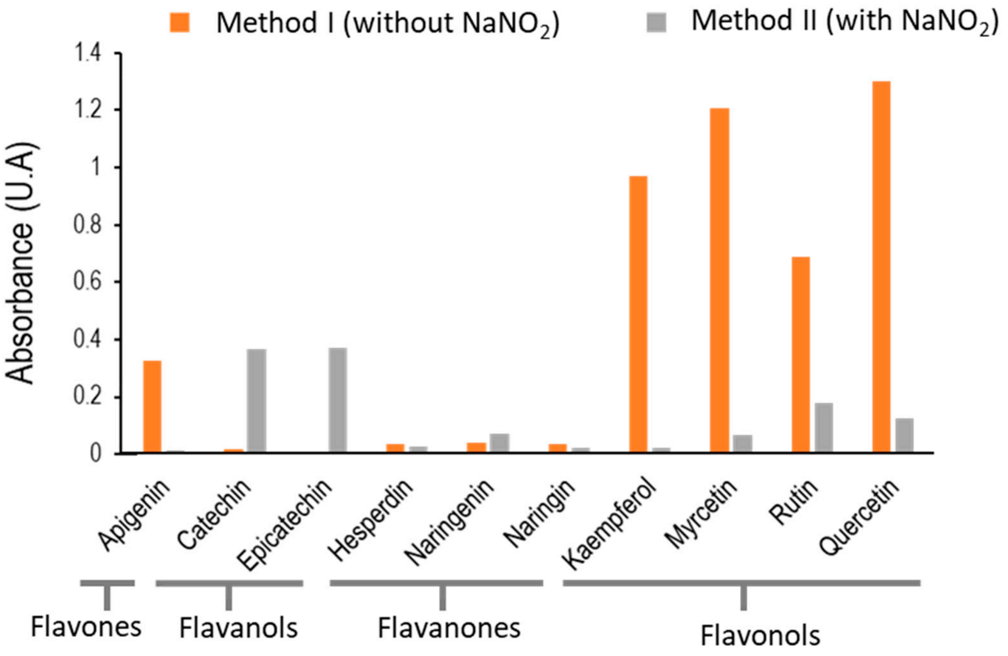

Figure S1. Comparison of the two aluminum complexation methods (with and without $NaNO_2$) using standards (500 mg L^{-1}) of compounds of different flavonoid subfamilies. Results are expressed as the absorbance measures at the corresponding wavelengths.

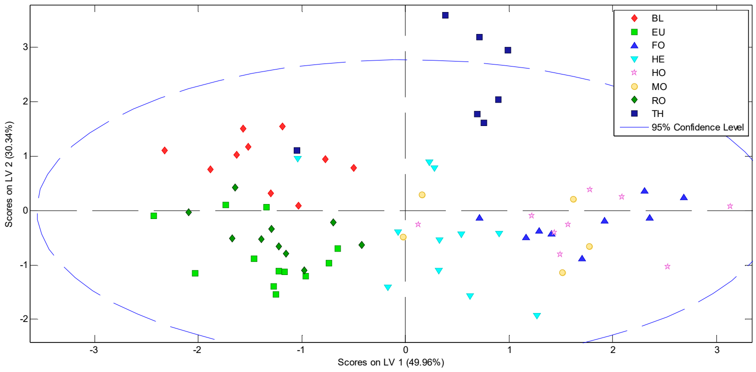

Figure S2. PLS-DA result for the multi-class classification of honeys according to their variety of origin. Three latent variables were used to build the model.

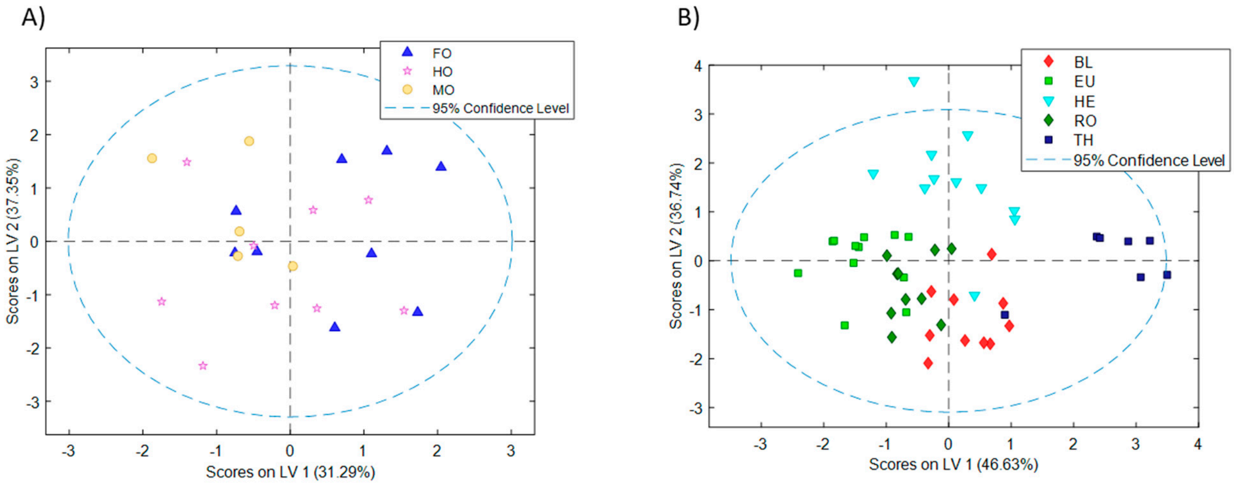

Figure S3. PLS-DA result for the multi-class classification of honeys according to their variety of origin. (A) honeydew honey and (B) blossom honeys. In both cases two latent variables were used to build the model.

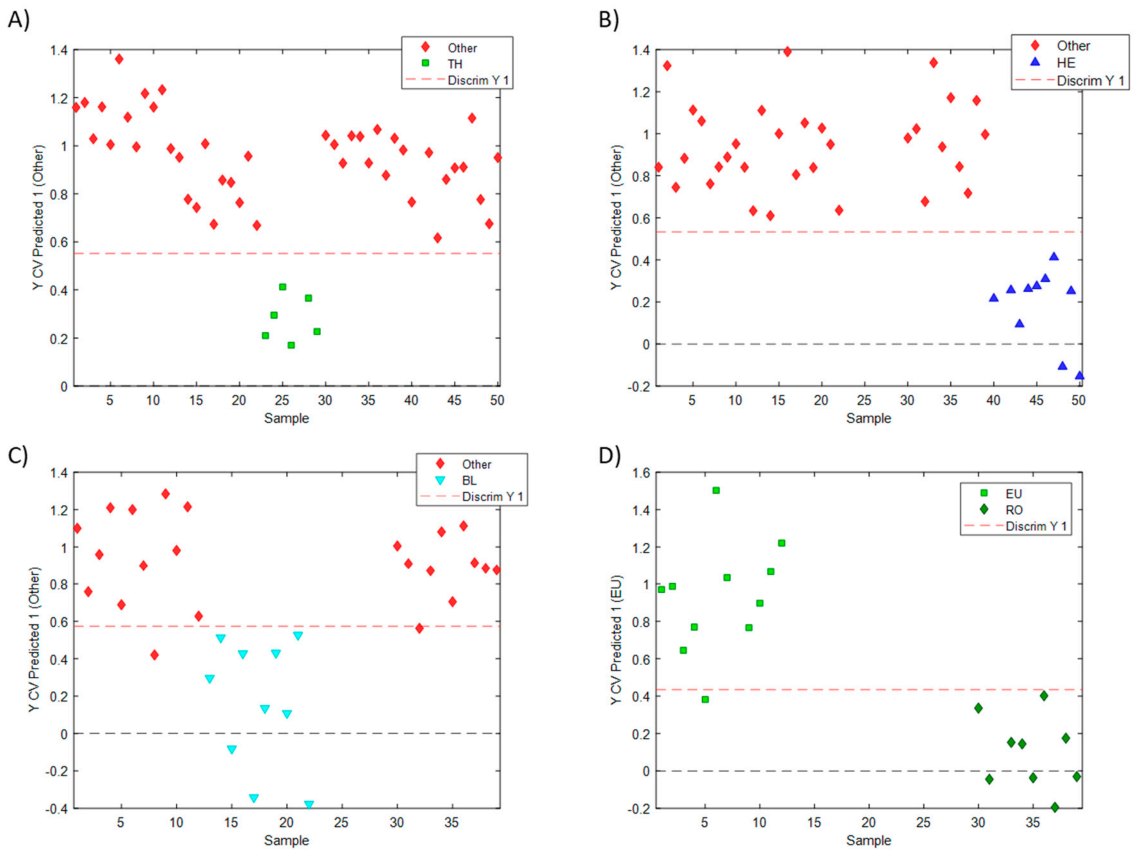

Figure S4. Two-class classification models obtained by PLS-DA according to the classification tree stablished. (A) thyme vs. others; (B) heather vs. others; (C) orange/lemon vs others; (D) eucaliptus vs. rosemary.

Table S1. Antioxidant indexes determined for the 73 samples under study.

Honey type	Variety	Code	FC index (mg eq gallic acid/g honey)	TFC (method I) (mg eq quercetin /g honey)	TFC (method II) (mg eq epicatechin /g honey)	FRAP (mg eq Trolox/g honey)
Blossom	Eucalyptus	**EU-1**	0.19	0.89	0.30	0.38
		**EU-2**	0.06	0.39	0.14	0.25
		**EU-3**	0.28	1.08	0.28	0.41
		**EU-4**	0.18	1.01	0.27	0.28
		**EU-5**	0.23	0.56	0.12	0.25
		**EU-6**	0.01	0.62	0.29	0.36
		**EU-7**	0.21	0.87	0.33	0.51
		**EU-8**	0.18	0.80	0.16	0.69
		**EU-9**	0.16	0.85	0.34	0.28
		**EU-10**	0.15	0.83	0.25	0.33
		**EU-11**	0.14	0.89	0.33	0.37
		**EU-12**	0.28	1.00	0.27	0.65
	**Mean ± SD**		0.17 ± 0.08	0.82 ± 0.20	0.26 ± 0.08	0.40 ± 0.15
	**Orange/lemon**	**BL-1**	0.22	0.34	0.07	0.50
		**BL-2**	0.35	1.47	0.03	0.68
		**BL-3**	0.32	0.40	ND	0.68
		**BL-4**	0.19	0.27	ND	0.30
		**BL-5**	0.36	0.54	ND	0.86
		**BL-6**	0.31	0.25	0.06	0.60
		**BL-7**	0.38	0.35	0.20	0.58
		**BL-8**	0.35	0.29	0.03	0.55
		**BL-9**	0.25	0.76	0.12	0.55
		**BL-10**	0.41	0.37	0.13	1.04
	**Mean ± SD**		0.32 ± 0.07	0.50 ± 0.37	0.06 ± 0.07	0.64 ± 0.20
	**Thyme**	**TH-1**	0.56	0.61	0.02	2.70
		**TH-2**	0.58	1.00	0.09	2.10
		**TH-3**	0.51	1.06	0.11	2.02
		**TH-4**	0.39	0.49	0.02	3.27
		**TH-5**	0.41	0.72	0.01	0.50
		**TH-6**	0.55	0.99	0.13	1.91
		**TH-7**	0.49	0.86	0.07	3.00
	**Mean ± SD**		0.50 ± 0.08	0.82 ± 0.22	0.07 ± 0.05	2.21 ± 0.92
	**Rosemary**	**RO-1**	0.28	0.79	0.22	0.17
		**RO-2**	0.32	0.39	0.23	0.17
		**RO-3**	0.31	1.08	0.19	0.58
		**RO-4**	0.23	0.23	0.13	0.05
		**RO-5**	0.31	0.70	0.24	0.15
		**RO-6**	0.32	0.61	0.05	0.06
		**RO-7**	0.30	0.75	0.30	0.28
		**RO-8**	0.44	1.04	0.22	0.22
		**RO-9**	0.28	0.21	0.23	0.16
		**RO-10**	0.34	0.50	0.20	0.18
	**Mean ± SD**		0.31 ± 0.05	0.63 ± 0.30	0.20 ± 0.07	0.20 ± 0.15
	**Heather**	**HE-1**	0.43	1.45	0.29	1.10
		**HE-2**	0.30	0.67	0.08	0.96
		**HE-3**	0.42	1.81	0.36	0.58
		**HE-4**	0.37	1.19	0.19	1.68
		**HE-5**	0.36	1.68	0.26	0.97
		**HE-6**	0.22	1.62	0.25	1.16
		**HE-7**	0.37	1.48	0.34	0.87
		**HE-8**	0.20	1.60	0.35	0.81
		**HE-9**	0.32	2.22	0.26	1.39
		**HE-10**	0.35	1.67	0.10	1.41
		**HE-11**	0.32	2.70	0.39	0.88
	**Mean ± SD**		0.33 ± 0.07	1.65 ± 0.52	0.26 ± 0.10	1.08 ± 0.32

Honey-dew	**Forest**	**FO-1**	0.65	1.94	0.34	0.97
		**FO-2**	0.81	2.55	0.20	1.34
		**FO-3**	0.62	2.56	0.27	1.62
		**FO-4**	0.52	2.92	0.18	1.22
		**FO-5**	0.48	2.36	0.24	1.18
		**FO-6**	0.60	2.38	0.16	0.35
		**FO-7**	0.82	1.93	0.23	1.38
		**FO-8**	0.69	1.47	0.28	0.87
		**FO-9**	0.67	1.23	0.22	0.57
		**Mean ± SD**	0.65 ± 0.11	2.15 ± 0.55	0.23 ± 0.06	1.06 ± 0.41
	**Holm oak**	**HO-1**	0.66	2.87	0.29	2.16
		**HO-2**	0.57	0.95	0.23	0.51
		**HO-3**	0.78	1.85	0.24	1.31
		**HO-4**	0.57	2.09	0.26	1.25
		**HO-5**	0.78	1.50	0.24	1.33
		**HO-6**	0.70	1.45	0.24	0.88
		**HO-7**	0.73	1.23	0.38	0.96
		**HO-8**	0.68	2.25	0.18	0.48
		**HO-9**	0.78	1.81	0.45	1.41
		**Mean ± SD**	0.70 ± 0.08	1.78 ± 0.58	0.28 ± 0.08	1.14 ± 0.52
	**Mountain**	**MO-1**	0.61	1.92	0.36	0.86
		**MO-2**	0.59	1.86	0.36	1.43
		**MO-3**	0.71	1.68	0.23	1.21
		**MO-4**	0.55	0.82	0.26	0.44
		**MO-5**	0.54	1.15	0.16	0.69
		**Mean ± SD**	0.60 ± 0.07	1.48 ± 0.48	0.27 ± 0.09	0.93 ± 0.40

Table S2. Results from two-class classification models obtained by PLS-DA. The calibration set comprises the 60% of all samples randomly chosen and the prediction set the 40% remaining samples.

Number of LV	Calibration		Validation		Prediction	
	Sensitivity	Specificity	Sensitivity	Specificity	Sensitivity	Specificity
2	1	1	1	0.92	0.90	1
